# Recurrent Stevens-Johnson Syndrome Following Inadvertent Re-exposure to Cotrimoxazole: A Preventable Adverse Drug Reaction

**DOI:** 10.7759/cureus.112317

**Published:** 2026-07-09

**Authors:** Niva Doshi, Shruti Brahmbhatt

**Affiliations:** 1 Department of Pharmacology, Shrimati Bhikhiben Kanjibhai Shah Medical Institute and Research Centre, Sumandeep Vidyapeeth Deemed to be University, Vadodara, IND

**Keywords:** adverse drug reaction, cotrimoxazole, recurrent stevens-johnson syndrome, scorten criteria, who-umc scale

## Abstract

Stevens-Johnson syndrome (SJS) is a severe cutaneous adverse drug reaction characterized by epidermal detachment and mucosal involvement. Cotrimoxazole is a well-known causative agent; however, recurrence following re-exposure remains underreported and preventable. We report a case of a 61-year-old female patient who presented with a burning sensation over the body, accompanied by superficial and crusted erosions over the lips and a few hyperpigmented macules on the chest for 10 days. On examination, a positive Nikolsky sign with <10% body surface area involvement was found, and a diagnosis of SJS was made. Notably, the patient had a documented history of SJS induced by cotrimoxazole five years earlier, with findings highly suggestive of recurrence following inadvertent re-exposure to the same causative agent. The disease severity was assessed using the SCORe of Toxic Epidermal Necrolysis (SCORTEN) criteria. The supportive treatment with dexamethasone, fluconazole, folic acid, and PCV transfusion was given, leading to gradual clinical improvement. This case highlights the importance of thorough drug-reaction history, avoidance of re-exposure to prevent recurrent severe cutaneous adverse reactions.

## Introduction

Cotrimoxazole (sulfamethoxazole + trimethoprim) belongs to the sulfonamide class of antimicrobial groups and is a fixed drug combination widely used for mild-to-moderate bacterial infections and as prophylaxis against opportunistic infections [1]. However, the use of co-trimoxazole is associated with hypersensitivity reactions in 1-3% of the general population; one form of hypersensitivity reaction is Stevens-Johnson syndrome (SJS) and toxic epidermal necrolysis (TEN) [2].

SJS and TEN are rare but life-threatening mucocutaneous reactions, with an incidence of 1-10 cases per million population per year [3]. They represent a spectrum of disease differentiated by the extent of epidermal detachment: SJS involves less than 10% of the body surface area (BSA), TEN involves more than 30%, and 10-30% involvement is classified as SJS-TEN overlap [4].

As the patient in this present case presented with less than 10% skin detachment, the diagnosis is confirmed as SJS.

Recurrent SJS due to inadvertent re-exposure to cotrimoxazole represents a preventable yet serious adverse drug reaction, emphasizing the importance of early recognition, strict drug avoidance, and effective patient counseling. However, there is limited literature focusing on preventable cases of recurrent SJS following re-exposure, particularly highlighting gaps in proper drug allergy documentation, patient awareness, and the need for improved pharmacovigilance. Therefore, this case report highlights a preventable recurrence of SJS and underscores the importance of early recognition, drug avoidance, and improved patient awareness.

## Case presentation

A 61-year-old female patient came to our hospital with the chief complaint of burning sensation over the upper and lower half of her body, with the development of superficial and crusted erosions over the upper and lower lip, and a few hyperpigmented macules over her chest since 10 days ago.

On examination, there was diffuse non-blanchable dusky erythema with peeling of skin and a few superficial erosions seen over the flexor aspect of the right and left forearms (Figure 1) and bilateral elbows. A few superficial erosions were present over the right buttock (Figure 2) and the lateral aspect of the right lower leg. Diffuse blanchable dusky erythema was on the left thigh, left knee, bilateral lower legs (Figure 3), and dorsum of the right foot. A single superficial ulcer with an erythematous base and hyperkeratotic margins is seen over the lateral aspect of the right foot. Severe hemorrhagic crusting and ulceration over both upper and lower lips are seen (Figure 4). No ocular or genital mucosal involvement was noted on examination. The Nikolsky sign was positive; tenderness was positive, and <10% BSA was involved. General examination showed all the vital parameters were within normal limits with no associated co-morbidities.

**Figure 1:**
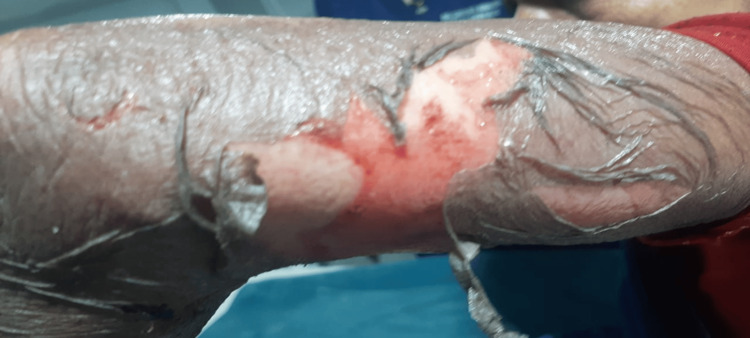
Non-blanchable erythema on the forearm Diffuse non-blanchable dusky erythema with superficial erosions over the flexor aspect of both forearms, photographed on Day 1 of admission (active disease phase, prior to initiation of systemic corticosteroid therapy). A positive Nikolsky sign was elicited in this region.

**Figure 2:**
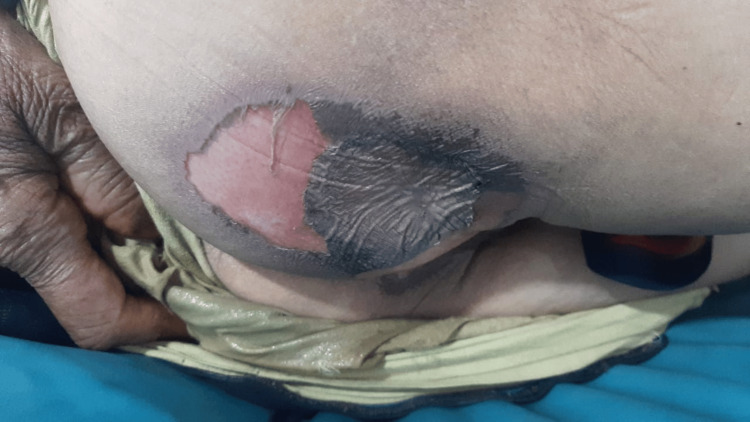
Superficial erosion over the right buttock, photographed on Day 1 of admission (active disease phase) Note the erythematous base consistent with early epidermal detachment involving less than 10% total body surface area.

**Figure 3:**
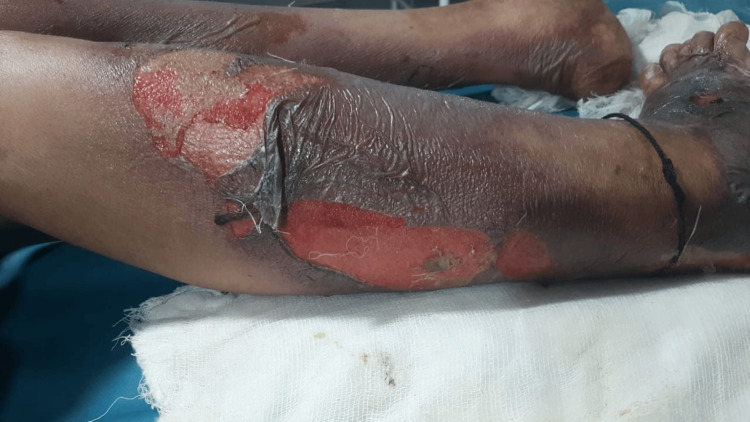
Blanchable erythema on the lower leg Diffuse blanchable dusky erythema over bilateral lower legs, photographed on Day 1 of admission (active disease phase, prior to treatment). Absence of epidermal detachment in this region is consistent with early inflammatory involvement without full-thickness necrosis.

**Figure 4:**
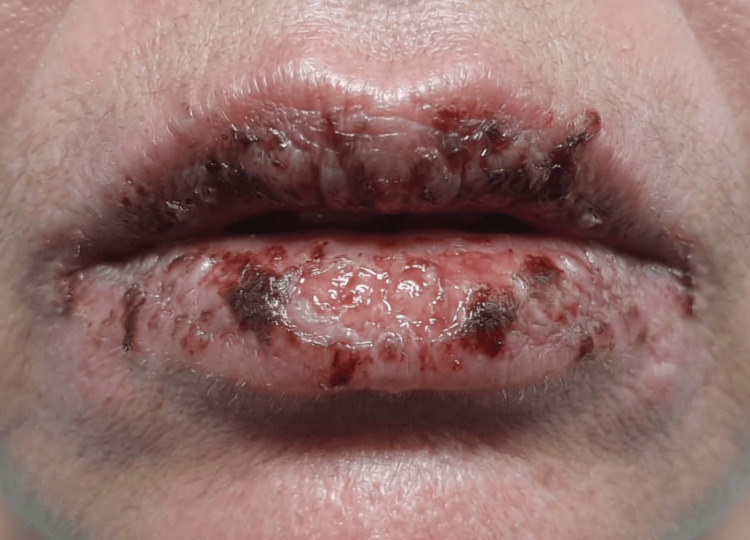
Severe hemorrhagic crusting with ulceration over both lips Mucosal involvement in the active phase (Day 1 of admission) showing severe erosive stomatitis with characteristic hemorrhagic crusting of the lips in a patient, representing early inflammatory involvement prior to treatment.

The patient had a history of high-grade fever with chills 10 days prior to admission. She initially sought treatment at a local clinic, where she was prescribed oral cotrimoxazole (trimethoprim 160 mg + sulfamethoxazole 800 mg) based on a culture sensitivity report positive for Enterobacter species. Notably, the patient had a documented history of Stevens-Johnson syndrome (SJS) due to cotrimoxazole five years earlier. However, during the consultation at the local clinic, the patient did not disclose this history and did not present prior medical documentation, resulting in inadvertent re-exposure to the offending drug. However, approximately 7-10 days after the initiation of cotrimoxazole, the patient developed similar cutaneous lesions, which progressively worsened, prompting her to present to our hospital for further evaluation and management. The document was reviewed at the time of admission; a copy of these records was not available for retention or inclusion in the current report.

A tablet of paracetamol (500 mg) was given concomitantly with cotrimoxazole for symptomatic fever management. However, paracetamol is not classified among the high-risk causative agents for SJS [5], and the onset of symptoms within 10 days of cotrimoxazole initiation, combined with the patient's documented prior SJS reaction to cotrimoxazole specifically, is consistent with cotrimoxazole as the sole causative agent.

The ALDEN (Algorithm of Drug Causality for Epidermal Necrolysis) score was used to determine the causality by establishing the temporal relationship between drug intake and the onset of symptoms [6], as shown in Table 1.

**Table 1:** ALDEN score Causality assessment was performed using the ALDEN (Algorithm of Drug Causality in Epidermal Necrolysis) score. Total scores were interpreted as the following: ≥6 (very probable), 4-5 (probable), 2-3 (possible), 0-1 (unlikely), and ≤-1 (very unlikely).

ALDEN Criteria	Cotrimoxazole	Paracetamol
Time from start to index day	+3 (7-28 days)	+1 (Not specific)
Drug present on index day	0 (Yes)	0 (Yes)
Pre-index day withdrawal	0 (No)	0 (No)
Risk of drug class	+3 (High risk)	0 (Low risk)
Other possible causes	+2 (No other plausible cause​​)	-1 (Strong alternative cause exists)
Previous reaction to the drug	+2 (Previous history present)	0
Total score	+10	0
Causality category	Very Probable	Very unlikely

On investigation, hemoglobin level was 8.7 g/dL (reference range: 11.7-15.3 g/dL), serum glucose level was 12 mmol/L (reference range: > 14 mmol/L), serum urea level was 11 mmol/L (reference range: > 10 mmol/L), and serum bicarbonate level was 21 mEq/L (reference range: < 20 mEq/L). The prognosis, assessed by the SCORTEN score (SCORe of Toxic Epidermal Necrolysis), was 2 at the time of admission, which indicated moderate disease severity with a predicted mortality of 12.1% [7].

The SCORTEN score for this patient is shown in Table 2.

**Table 2:** The SCORTEN score SCORTEN (Severity-of-Illness Score for Toxic Epidermal Necrolysis) was calculated on admission to assess disease severity and predict mortality. One point was assigned for each positive parameter. Total SCORTEN ranges from 0-7, with higher scores indicating increased mortality risk (0-1: low risk, 2: ~12% mortality, 3: ~35%, 4: ~58%, ≥5: >90%).

SCORTEN Parameter	Clinical/Laboratory Findings	Points
Age > 40 years	61 years	1
Heart Rate > 120 bpm	Hemodynamically stable	0
Malignancy	No associated co-morbidities	0
Body Surface Area (BSA) Detachment > 10%	< 10% BSA involved	0
Serum Urea > 28 mg/dL (10 mmol/L)	11 mmol/L	1
Serum Glucose > 252 mg/dL (14 mmol/L)	12 mmol/L	0
Serum Bicarbonate < 20 mEq/L	21 mEq/L	0
TOTAL SCORTEN		2

Tablet cotrimoxazole was stopped immediately, and the patient was treated with an 8 mg intravenous (IV) twice-daily dexamethasone with tapering dose for 11 days. This decision was taken considering the early inflammatory phase of the disease and limited body surface area involvement at presentation. The patient was closely monitored for secondary bacterial infection throughout the course of corticosteroid therapy, with no infectious complications observed during the hospital stay. Oral fluconazole was administered prophylactically in view of extensive mucosal involvement, prolonged systemic corticosteroid use, and the patient’s relatively immunocompromised state during acute illness--all recognized risk factors for opportunistic fungal infection in SJS. Packed cell volume transfusion was given for anemia to improve oxygen delivery and overall recovery. Additionally, topical beclomethasone dipropionate and fusidic acid cream were used for oral mucosa, and Vaseline jelly was used for skin lesions. The patient showed gradual clinical improvement, with drying of lesions and resolution of tenderness and absence of new blister formation, and was discharged after 11 days of hospital stay. On follow-up, there were no residual sequelae, including cutaneous, mucosal, or ocular complications.

## Discussion

SJS is a severe, life-threatening skin and mucous membrane disorder marked by widespread epidermal detachment, mucosal erosion, and a positive Nikolsky sign (skin separation upon light friction). It originates as a delayed (Type IV) hypersensitivity reaction, usually triggered by medications, where the immune system, specifically cytotoxic T cells and natural killer (NK) cells, becomes activated and attacks keratinocytes. These immune cells release toxic proteins, namely, granulysin, granzyme B, and perforin, which trigger extensive cell death (apoptosis) and lead to widespread tissue destruction [8].

The SCORTEN scoring system is used to evaluate the severity of the disease in SJS, which predicts mortality risk by analyzing specific clinical and laboratory parameters. Upon admission, this patient’s SCORTEN score indicated moderate severity, necessitating close monitoring and supportive management [9].

Cotrimoxazole is a well-recognized trigger for SJS/TEN and is one of the most common causative agents reported in India. The onset typically ranges from 4 to 28 days after drug initiation, which is consistent with the timeline observed in the present patient [8].

This case is particularly notable because its findings are highly suggestive of recurrence of SJS following inadvertent re-exposure to cotrimoxazole five years after an initial reaction. The prior episode was verified through available documentation reviewed at our hospital, although a copy of the records was not available for retention. However, during a subsequent consultation at a local clinic, the patient did not disclose this history, leading to the inadvertent re-prescription of cotrimoxazole. The clinical timeline and presentation are consistent with a rechallenge-induced event. Such re-exposure often triggers a faster, more intense immune response due to immunological memory, highlighting that these adverse events are preventable [10].

This case also exposes critical system-level failures in medication safety. The patient had a documented history of cotrimoxazole-induced SJS five years prior, yet the drug was re-prescribed without apparent awareness of this allergy. This raises important questions regarding allergy documentation practices, the reliability of electronic or paper-based alert systems, and the adequacy of medication reconciliation at the time of prescribing. It is equally possible that the allergy was not recorded in an accessible or standardized format or that the patient was not adequately counselled after her first episode about the lifelong risk of re-exposure. From a pharmacovigilance perspective, this constitutes a serious and avoidable medication error; one that could have been avoided with robust allergy documentation, proper history taking, and systematic patient education. Clinicians must recognize that a history of SJS constitutes an absolute contraindication to re-exposure to the offending drug, and healthcare systems must support this through reliable, accessible allergy recording mechanisms. This brings to the fore the fundamental significance of meticulous drug-reaction history, proper documentation, and patient education to avoid inadvertent re-administration.

Documented cases of SJS following confirmed re-exposure to cotrimoxazole are exceedingly rare. According to Matei et al., the reintroduction of cotrimoxazole in a previously sensitized patient resulted in the recurrence of severe cutaneous adverse reactions [11].

Earlier reviews also highlighted that rechallenge with cotrimoxazole can provoke a more rapid and severe immune-mediated response due to immunological memory [12,13]. These observations reinforce the preventable nature of rechallenge-associated SJS and the critical need for strict drug avoidance in the future.

According to Mockenhaupt et al., sulfonamide antibiotics rank among the top three drug groups implicated in SJS/TEN globally, alongside antiepileptic drugs and allopurinol [14].

Cross-reactivity is another clinically relevant aspect. Hypersensitivity reactions to cotrimoxazole are primarily attributed to the arylamine group in sulfonamide antibiotics, which undergoes metabolic activation leading to reactive intermediates and immune-mediated cytotoxicity. Patients with prior reactions to sulfonamide antibiotics are at increased risk of recurrence upon re-exposure, as likely observed in the present case. However, comparative literature, including a large cohort study by Strom et al., suggests that cross-reactivity between sulfonamide antibiotics and non-antibiotic sulfonamides (e.g., thiazide diuretics, sulfonylureas) is minimal due to structural differences. This distinction is important to avoid unnecessary restriction of therapeutically useful medications [15]. Similar cases have been reported with non-antibiotic drugs such as febuxostat (Sidhu et al.) [16], emphasizing that SJS/TEN is associated with multiple high-risk drug classes, including antibiotics, antiepileptics, and urate-lowering agents.

Reports of recurrent SJS following confirmed re-exposure to cotrimoxazole are highly uncommon. Most of the existing literature is limited to isolated case reports and narrative reviews rather than well-documented rechallenge cases. This highlights both the rarity of such events and the clinical importance of the present case.

The ALDEN score was calculated to be +10, which indicates a very probable causal relationship between cotrimoxazole and the development of Stevens-Johnson syndrome in the present case [6]. Additionally, the adverse drug reaction was classified under the moderate category according to the Modified Hartwig’s Scale, as it requires the immediate discontinuation of the drug due to its potential to cause more permanent damage to skin and soft tissue [17].

## Conclusions

This case highlights a preventable yet potentially life-threatening adverse drug reaction--recurrent Stevens-Johnson syndrome (SJS) following inadvertent re-exposure to cotrimoxazole. The findings are highly suggestive of a rechallenge-induced recurrence in a patient with a documented prior history of the same reaction. The report accentuates the importance of a thorough drug-reaction history, proper documentation, and strict avoidance of rechallenge with culprit medications. Healthcare institutions should ensure that severe drug reactions, such as SJS, are permanently flagged in patient records and that allergy information is routinely reviewed as part of medication reconciliation before prescribing. Patients must be clearly counselled at the time of the initial SJS episode regarding the absolute contraindication to re-exposure to the offending agent, and this information should be made available across all points of care. Early recognition, prompt withdrawal of the offending drug, and timely supportive management are key determinants of favorable outcomes in SJS. Reporting such cases strengthens pharmacovigilance efforts and reinforces awareness among clinicians regarding severe cutaneous adverse drug reactions.
